# An investigation of scale effects in family substance abuse treatment programs

**DOI:** 10.1186/1747-597X-5-14

**Published:** 2010-07-05

**Authors:** A James Lee

**Affiliations:** 1 Department of Community Health and Sustainability, School of Health and Environment, University of Massachusetts Lowell, 300 Weed Hall, 3 Solomont Way Lowell, MA 01854, USA

## Abstract

This short report investigates scale effects in family substance abuse treatment programs. In Massachusetts, the family substance abuse treatment programs were much more costly than other adult residential treatment models. State officials were concerned that the "scale" or size of these programs (averaging just eight families) was too small to be economical. Although the sample size (just nine programs) was too small to permit reliable inference, the data clearly signalled the importance of "scale effects" in these family substance abuse treatment programs. To further investigate scale effects in family substance abuse treatment programs, data from the Center for Substance Abuse Treatment's (CSAT's) Residential Women and Children and Pregnant and Postpartum Women (RWC-PPW) Demonstration were re-analyzed, focusing on the relationship between cost per family-day and the estimated average family census. This analysis indicates strong economies of scale up until an average family census of about 14, and less apparent scale effects beyond that point. In consideration of these and other study findings, a multidisciplinary interagency team redesigned the Massachusetts' family treatment program model. The new programs are larger than the former family treatment programs, with each new program having capacity to treat 11 to 15 families depending on family makeup.

## Short report

The Massachusetts Bureau of Substance Abuse Services (BSAS) contracts with licensed not-for-profit vendors to provide residential rehabilitation services to individuals and families recovering from addiction to alcohol and other drugs [1]. In FY2002, BSAS funded nine family treatment programs, ranging in size from five to 12 families. The programs provided shelter and coordinated substance abuse treatment on behalf of predominantly female-headed "homeless" families. The women had long-term substance abuse problems, and many referrals were involuntary. The programs themselves provided case management services and some "life skills" education, but most individual and group counseling was provided on a fee-for service basis and paid separately by Medicaid and others.

In conducting this study, we collected and analyzed the program-specific Schedule B reports included in the audited FY 2002 Uniform Financial Reports (UFRs) submitted by the parent organizations of all nine family treatment programs. The UFRs provided a reasonably authoritative source for facility cost and statistical information. In addition, we conducted in-depth site visit interviews with eight of the nine programs and collected additional, substantially qualitative information on program operations.

The nine family treatment programs had a total capacity of 73 families, which is, an average of just 8.1 families per program. The programs' average lengths-of-stay varied from 104 days to 215 days, and the program average length-of-stay was 176 days (nearly half a year). Moreover, for those who actually completed treatment (and did not leave against advice), the program average length-of stay was 331 days (nearly a year), and ranged from 200 days to 492 days. The number of children per family (i.e., the child-to-family ratio) varied from 1.1 to 2.2, and averaged 1.6

Averaging over the nine programs, the average total cost per family-day (counting only adults) was $224, and the total cost per family-day varied from $157 to $312. Excluding program and administrative expense, the direct care cost per family-day averaged $159, varying from $108 to $207 per family-day. The average total cost per person-day was $86, varying from $55 to $115 per person-day; and the average direct care cost per person-day was $61, varying from $38 to $76 per person-day.

Since the administrative and facility costs are largely uncontrollable at the program level, this direct care cost measure more fairly reflects differences in program cost structure (and arguably program efficiency). It includes the cost of all on-site program personnel, any professional service contracting, groceries and other supplies expense.

**Figure 1:**
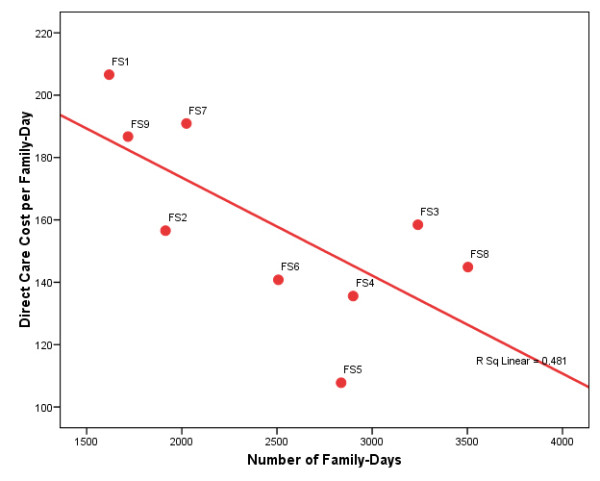
**Plot of Direct Care Cost per Family-Day Vs. Total Family-Days**. Massachusetts Family Treatment Providers, FY 2002 (N = 9). Source: Program-specific Schedule B's included in FY 2002 Uniform Financial Reports submitted to the Executive Office of Health and Human Services.

**Figure 2:**
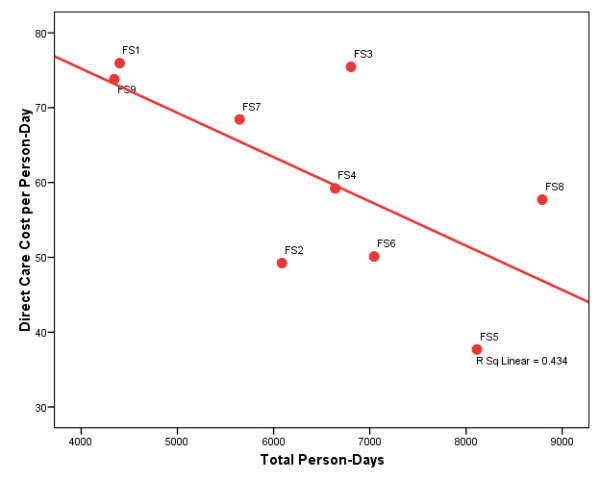
**Plot of Direct Care Cost per Person-Day Vs. Total Person-Days**. Massachusetts Family Treatment Providers, FY 2002 (N = 9). Source: Program-specific Schedule B's included in FY 2002 Uniform Financial Reports submitted to the Executive Office of Health and Human Services.

Now what accounts for these cost differences across programs? Could it be the scale differences in these comparatively small programs? In Figure 1, the direct care cost per family-day is plotted against the number of family-days. The downward sloping line represents the best bivariate linear fit to the data. As we see, a simple linear regression model suggests that the direct care costs fall pretty sharply as client volume increases. In Figure 2, the direct care cost per person-day is similarly plotted against the number of person-days, and the result is much the same. The bivariate regression line likewise suggests that the direct care costs decline quickly as client volume increases. In both cases, the right-hand-side scale variables are significant at the 0.05 level or better.

How confident can we be that we are accurately modeling costs per day and that the model correctly indicates scale effects? As the reader can plainly see from Figures 1 and 2, the fit to the data is not tight. So what else might account for the cost variances? Adding just one variable to these models--the child-to-family ratio--dramatically improves the goodness of fit. The percentage of variance explained in the direct care cost per family-day model increases from 48 percent to 68 percent, and the percentage of variance explained in the direct care cost per person-day model increases from 43 percent to 80 percent. Moreover, both variables in these models are significant at the 0.10 level or better, and the volume relationships are essentially unchanged from those depicted in Figures 1 and 2. Indeed, the person-day model predicts that increasing the average program size by just three families would reduce the average direct care cost by 24 percent, from $61 per person-day to $46 per person-day. The findings are little different if looking at total cost per family-day or total cost per person-day (i.e., also including the program and administrative costs). Although the sample size (just nine observations) is too small to permit reliable inference, the findings clearly signal the importance of "scale effects" in family substance abuse treatment programs.

Two recent studies--Beaston-Blaakman, Shepard, Horgan and Ritter [2] and: Duffy, Dunlap, Feder and Zarkin [3]--report finding substantial scale economies in outpatient substance abuse treatment. However, little evidence of systematic efforts to investigate scale effects in family and other residential substance abuse treatment programs is found in the literature. One study [4] analyzed data from the RWC-PPW Demonstration [5] and found fairly compelling evidence for the cost advantages of increasing program size. In particular, Harwood, Kallinis and Liu [4] conducted a series of regression analyses of the relationship between cost and scale in the RWC-PPW Demonstration. The executive summary of this report concludes (p. i), "This analysis demonstrates that larger residential substance abuse providers, on average, have lower costs per day than smaller providers." The empirical relationship is a strong one. For the RWC programs, their analyses indicate that a 10 percent increase in clients would be associated with about a 6 percent decrease in cost per client day. Moreover, the report concludes (p. 26) that, "Small providers should be conscious of potential benefits that could be realized from growth or merger/consolidation with other providers." While undeniably germane, these findings are nevertheless somewhat difficult to generalize to the family treatment programs because the analysts focus on the relationship between cost per person-day and the average total census (i.e., counting both women and children).

To further investigate scale effects in family substance abuse treatment programs, we conducted a new analysis of the RWC-PPW Demonstration data, focusing on the relationship between cost per family-day and the estimated average family census. For this purpose, we used the demonstration data as included in the 2000 N-SSATS public use data file [6]. Although the public use data file does not include a bed-size variable, it includes information sufficient to estimate the average daily family census. For the 22 RWC programs reporting in 1997, the estimated average family census was 14, and the estimated median was 11. The estimated average daily family census ranged from a low of 6 to a high of 43. Only two programs had an average family census larger than 20. Of course, the program bed-sizes would have been somewhat larger.

**Figure 3:**
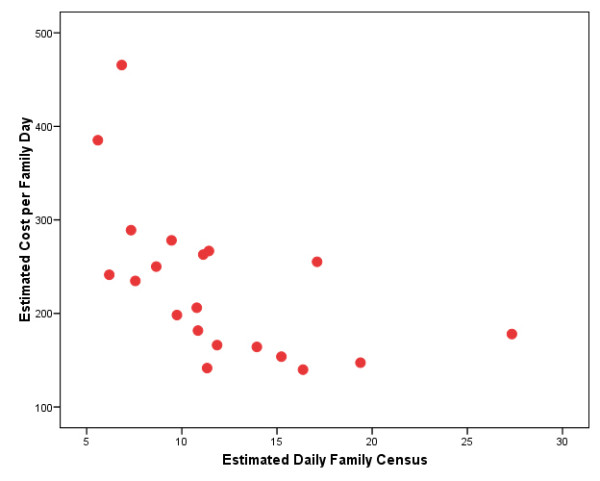
**Plot of Cost per Family-Day Vs. Daily Family Census**. Residential Women and Children (RWC) Programs, 1997 (N = 20)

The RWC-PPW Demonstration data were re-analyzed, focusing on the relationship between cost per family-day and estimated average family census. This relationship is depicted in Figure 3 for the 20 RWC programs with a daily family census of 30 or less in 1997. It was thought unlikely that anyone in Massachusetts would choose to develop a program with capacity for more than 30 families. A simple linear regression model accounts for approximately one-third of the variance in cost per family day, and the family census variable is significant at the 0.01 level. The data clearly indicate scale effects. Although the sample size is too small to fit a non-linear model, the data scatter in Figure 3 nevertheless suggests that scale effects quickly plateau. In particular, for five of the six programs with a family census of approximately 14 or more, the estimated costs per family-day are similar, varying only from $140 to $178 per family-day, and averaging $157. The average cost per family-day for the 14 smaller programs is $255.

**Figure 4:**
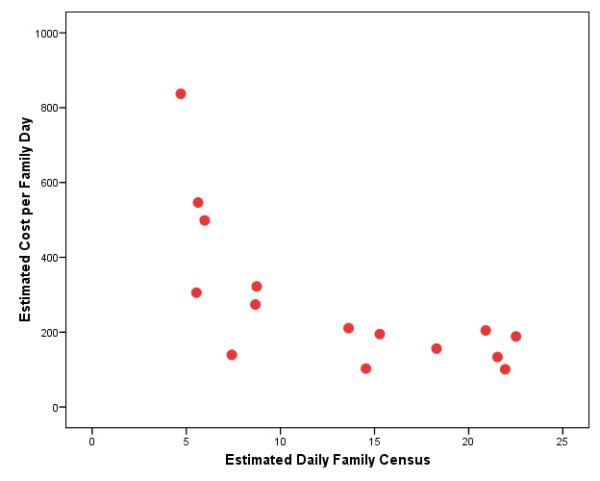
**Plot of Cost per Family-Day Vs. Daily Family Census**. Pregnant and Postpartum Women (PPW) Programs, 1997 (N = 15)

For the 16 PPW programs that reported in 1997, the estimated average family census was 15 and the median was 14. The estimated average daily family census ranged from a low of 5 to a high of 50. Five programs had an average family census larger than 20. Figure 4 similarly depicts the relationship between cost per family-day and average daily family census in the 15 PPW programs with a daily family census of 30 or less in 1997. It indicates a pattern remarkably similar to that in Figure 3. In particular, it similarly indicates that the family-day cost initially falls rather sharply with program size and then seems to plateau once an average family census of 14 or so is reached. The estimated costs per family-day are little more than $200 or less for all seven programs with a family census of 14 or more. For these larger programs, the cost-per-family days averages $152, compared to $392 for the eight smaller programs.

In summary, our re-analysis of CSAT's RWC-PPW demonstration cost data finds strong economies of scale up until an average family census of about 14. It is important to acknowledge, however, that this and other cost findings reported herein are altogether unadjusted for case mix differences. Nor did we have any outcome data available to similarly investigate the relationship between cost and outcomes.

In consideration of the cost analysis and other study findings, a multidisciplinary interagency team redesigned the Commonwealth's family treatment program model. In 2005, BSAS introduced a new modality, Family Residential Substance Abuse Treatment Services, to replace both its costly family treatment programs and its underutilized Special Residential Services for Women (and children) programs. These new programs are larger than the former family treatment programs, with each new program having capacity to treat 11 to 15 families depending on family makeup. Eight such programs were funded at an average cost of no more than $162 to $221 per family-day, and this included the cost of treatment services formerly paid by Medicaid but now provided by the programs themselves. The new modality also included a variety of clinical enhancements, namely, improved outcome monitoring and intensive case management services.

## Competing interests

The author declares that they have no competing interests.

## Author information

**A. James Lee**, Ph.D., is Associate Professor within the Department of Community Health and Sustainability, School of Health and Environment, University of Massachusetts at Lowell (Lowell, Massachusetts). He is a healthcare economist.
